# GSK2334470 attenuates high salt-exacerbated rheumatoid arthritis progression by restoring Th17/Treg homeostasis

**DOI:** 10.1016/j.isci.2024.109798

**Published:** 2024-04-26

**Authors:** Qian Mo, Mansoor Bolideei, Shan-Jie Rong, Jia-Hui Luo, Chun-Liang Yang, Wan-Ying Lu, Qi-Jie Chen, Jia-Wei Zhao, Fa-Xi Wang, Ting Wang, Yang Li, Xi Luo, Shu Zhang, Fei Xiong, Qi-Lin Yu, Zi-Yun Zhang, Shi-Wei Liu, Fei Sun, Ling-Li Dong, Cong-Yi Wang

**Affiliations:** 1 Department of Respiratory and Critical Care Medicine, the Center for Biomedical Research, NHC Key Laboratory of Respiratory Diseases, Tongji Hospital, Tongji Medical College, Huazhong University of Sciences and Technology, Wuhan, China; 2 Department of Rheumatology, Tongji Hospital, Huazhong University of Science and Technology, Wuhan, China; 3 Cancer Center, Renmin Hospital of Wuhan University, Wuhan, China; 4 Shanxi Bethune Hospital, Shanxi Academy of Medical Science, Tongji Shanxi Hospital, Third Hospital of Shanxi Medical University, the Key Laboratory of Endocrine and Metabolic Diseases of Shanxi Province, Taiyuan, China

**Keywords:** Pharmacology, Natural sciences, Biological sciences, Physiology, Immunology

## Abstract

High salt (HS) consumption is a risk factor for multiple autoimmune disorders via disturbing immune homeostasis. Nevertheless, the exact mechanisms by which HS exacerbates rheumatoid arthritis (RA) pathogenesis remain poorly defined. Herein, we found that heightened phosphorylation of PDPK1 and SGK1 upon HS exposure attenuated FoxO1 expression to enhance the glycolytic capacity of CD4 T cells, resulting in strengthened Th17 but compromised Treg program. GSK2334470 (GSK), a dual PDPK1/SGK1 inhibitor, effectively mitigated the HS-induced enhancement in glycolytic capacity and the overproduction of IL-17A. Therefore, administration of GSK markedly alleviated HS-exacerbated RA progression in collagen-induced arthritis (CIA) model. Collectively, our data indicate that HS consumption subverts Th17/Treg homeostasis through the PDPK1-SGK1-FoxO1 signaling, while GSK could be a viable drug against RA progression in clinical settings.

## Highlights

• High-salt (HS) intake positively correlates with RA severity
• HS exacerbates Th17/Treg imbalance via PDPK1-SGK1-FoxO1 signaling
• GSK rectifies HS-induced glycolytic reprogramming and Th17/Treg imbalance
• GSK mitigates HS-exacerbated RA progression

## Graphical abstract

**Figure:**
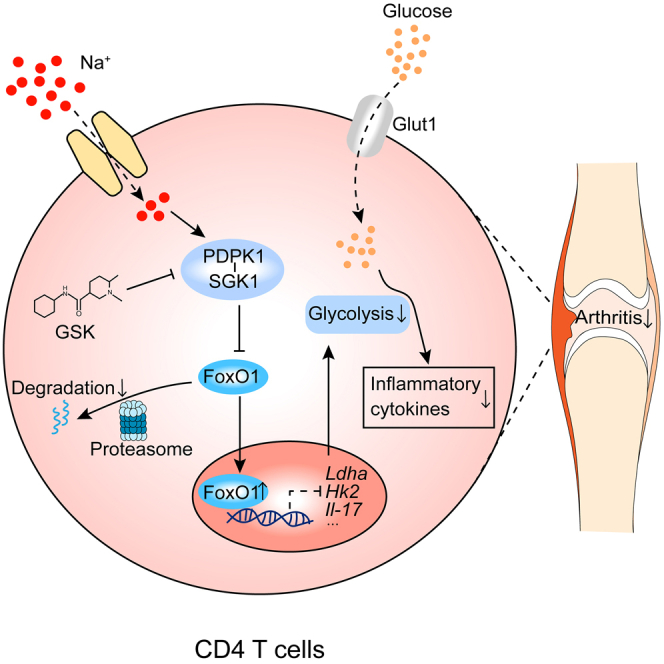

## Introduction

Rheumatoid arthritis (RA) is a chronic autoimmune disorder characterized by the symmetrical and progressive joint inflammation, ultimately leading to the degradation of articular cartilage, bone erosion and physical disability.^1^ The etiology of RA involves genetic predisposition as well as risk factors including cigarette smoking, obesity, microbial infections and so on.^2^ High salt (HS) diet has been acknowledged as a potential contributing factor for autoimmune diseases such as inflammatory bowel disease (IBD), experimental autoimmune encephalomyelitis (EAE), and systemic lupus erythematosus (SLE).^3^^,^^4^^,^^5^^,^^6^^,^^7^ Under the RA setting, elevated sodium intake in smokers has been linked to an increased likelihood of anti-citrullinated protein antibody (ACPA) positivity and the occurrence of RA. Additionally, RA patients presenting with bone erosion at the time of their initial diagnosis possess higher levels of sodium excretion compared to those patients absent of bone erosion.^8^^,^^9^^,^^10^^,^^11^ These preliminary clinical studies on RA patients collectively supported the notion of “salty truth” in autoimmune disorders.^12^ Nonetheless, compared to other disease conditions, the phenotypes and specific mechanisms linking salt consumption to RA severity are insufficiently addressed. Knowledge gaps exist regarding: (1) whether and how HS diet modifies RA progression in genetic predisposed individuals, (2) molecular consequences of HS intake among RA patients, and most critically (3) the pursuit of promising drug targets that can also be applied to RA patients who prefer salty taste. Considering the potency of dietary intervention in RA management^13^ and the prevalence of high-salt containing food in nowadays society, filling those knowledge gaps could lead to novel dietary recommendations and therapeutic strategies against RA, making this area of research scientifically vital and clinically relevant.

The immunopathology of RA is underscored by the imbalance between T helper 17 (Th17) cells and regulatory T (Treg) cells. Th17 cells instigate joint inflammation via releasing an array of pro-inflammatory cytokines such as tumor necrosis factor-alpha (TNF-α), interleukin (IL)-17A and granulocyte-macrophage colony-stimulating factor (GM-CSF). IL-17A stimulates fibroblasts to produce receptor activator od nuclear factor-kappa B ligand (RANKL), which in turn promotes osteoclast maturation and the secretion of destructive enzymes like matrix metalloproteinase (MMP)-1 and MMP-3.^14^ Elevated levels of IL-17A in the serum and synovial fluid of RA patients positively correlate with autoantibody titer and disease activity.^15^^,^^16^^,^^17^^,^^18^ In sharp contrast, Treg cells are pivotal negative regulators in RA progression and a major source of anti-inflammatory IL-10. Unlike wild-type mice, *Il-10* deficient mice develop more severe arthritis following type II collagen induction.^19^^,^^20^^,^^21^ Consistently, sodium butyrate inhibits collagen-induced arthritis (CIA) by enhancing IL-10 expression and Treg cell polarization, while anti-IL-10 neutralizing antibodies abolish the beneficial effects of sodium butyrate.^22^ A decline in the number or functionality of Treg cells leads to an exaggerated Th17 response, resulting in enhanced autoimmune reaction and joint inflammation.^19^^,^^20^^,^^21^^,^^23^ Worse still, Treg cells within RA patients tend to convert into pathogenic Th17 cells and accumulate in the inflammatory synovial membrane, which renders a Th17/Treg imbalance along with exacerbated disease progression.^24^ In animal models, the ablation of Th17 cells or their associated cytokines alleviates arthritis symptoms,^25^^,^^26^ while depletion of Treg cells or IL-10 exacerbates RA severity.^19^^,^^20^^,^^27^ Therefore, an imbalance between Th17 and Treg cells is crucial in RA initiation and progression.

Glucose metabolism has been identified as a master regulator of T cell function and differentiation.^28^ While Th17 and effector T cells mainly rely on aerobic glycolysis for energy demand, Treg cells and memory T cells depend on mitochondrial respiration and fatty acid oxidation.^29^ The serine/threonine kinase 3′-phosphoinositide dependent kinase 1 (PDPK1) is closely associated with glycolytic metabolism and acts as a key regulator of cell growth and proliferation.^30^^,^^31^ In CD4 T cells, PDPK1 integrates signals from the T cell receptor (TCR) and CD28, thereby participating in the activation of nuclear transcription factor-kappa B (NF-kB) signaling pathway.^32^ PDPK1 is also essential for the transition of thymocytes from the double-negative (DN) stage to the double-positive (DP) stage. However, its absence does not affect the thymic development of DP cells into single-positive (SP) cells.^33^ In rats with induced arthritis, PDPK1 overexpression accelerates disease progression by promoting fibroblast-like synoviocyte (FLS) proliferation.^34^ Conversely, inhibition of PDPK1 restrains the invasive capacity of human FLSs via reducing the expression of MMP-2 and MMP-9.^35^^,^^36^ These lines of evidence indicate that PDPK1 participates in RA progression through regulating the activity of FLS, yet its role in T cell immunometabolism in the context of RA remains unexplored.

PDPK1 activates several kinases of the protein kinase A-protein kinase G-protein kinase C (AGC) family, notably including serum- and glucocorticoid-induced protein kinase 1 (SGK1).^30^ SGK1 acts as a “sodium sensor” in T cells in response to extracellular salt fluctuation.^37^^,^^38^^,^^39^^,^^40^ This is biologically significant as SGK1 plays a critical part in IL-23R mediated inhibition of Treg cells and the promotion of Th17 program, both of which are pivotally engaged in the immunopathology of RA.^41^ The rationale of our study hinges on these observations and we determined to elucidate the mechanisms whereby increased salt intake exacerbates RA, with a focus on the PDPK1/SGK1 pathway. HS intake is operationally defined here as a daily consumption of sodium chloride exceeding 5 g, as the World Health Organization (WHO) recommends no more than 5 g daily salt intake, a threshold we adopted to categorize RA patients into low and HS diet groups based on their self-assessment.^42^ In accordance, we assess the potential of utilizing the dual PDPK1/SGK1 inhibitor, GSK2334470 (referred to as GSK hereafter), as a novel drug for RA treatment. Our study thus provides valuable insight into how HS diet modifies RA progression and offers a promising therapeutic strategy for RA treatment.

## Results

### The phosphorylated PDPK1 and SGK1 in CD4 T cells correlate with RA severity

**Figure 1:**
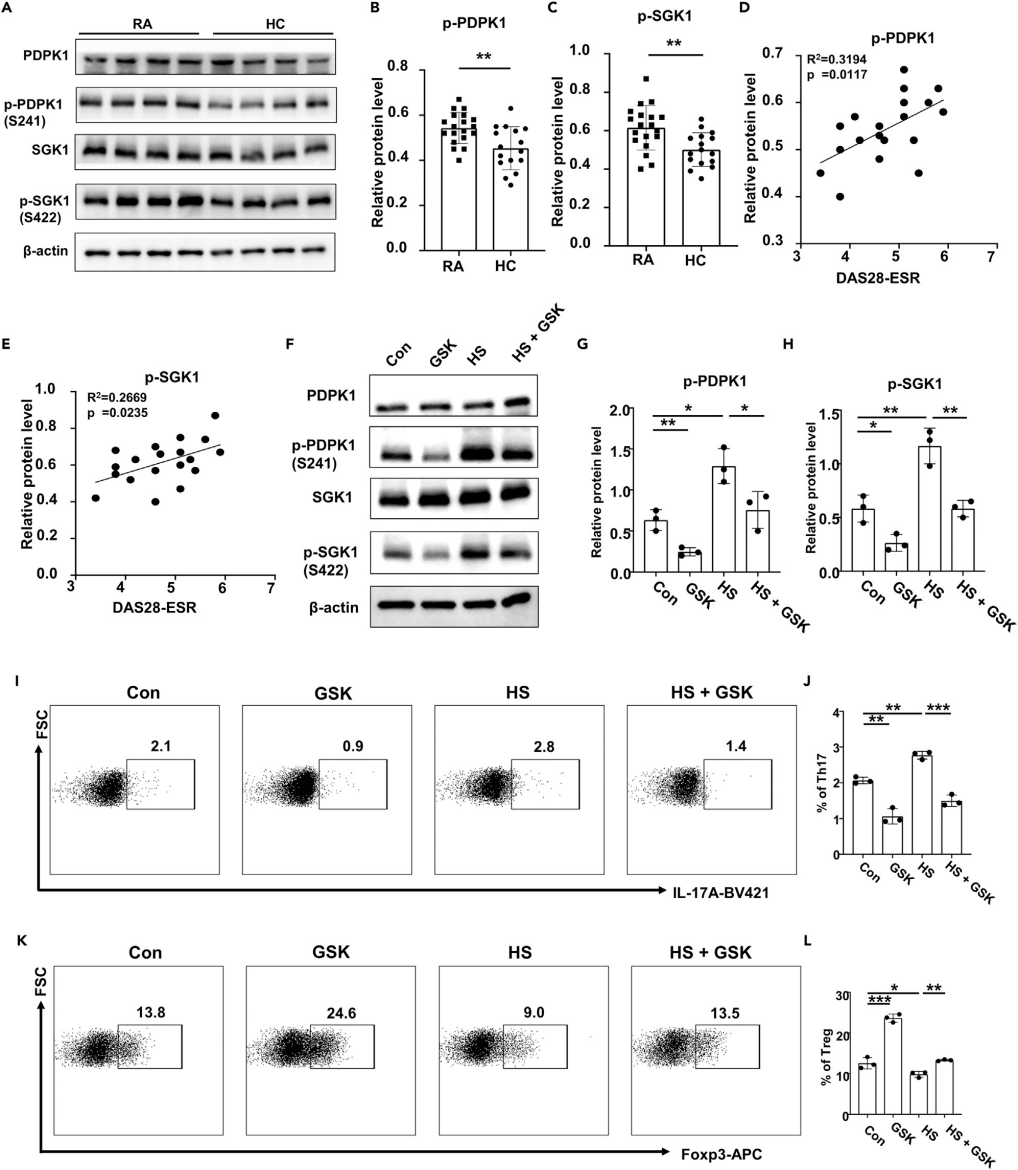
Phosphorylation levels of PDPK1 and SGK1 in CD4 T cells correlate with RA severity (A) Expression levels of (p-)PDPK1 and (p-)SGK1 in CD4 T cells of RA (*n* = 19) and HC (*n* = 16) groups were detected by Western blot. (B and C) Quantification of the phosphorylation levels of PDPK1 and SGK1 in CD4 T cells of RA and HC groups. (D and E) Correlation analysis of disease activity index DAS28-ESR with the phosphorylation levels of PDPK1 and SGK1. (F–H) CD4 T cells were isolated from RA patients and cultured for 3 days in the presence of HS (40 mmol/L) and/or GSK (2.5 μmol/L). Expression levels of (p-)PDPK1 and (p-)SGK1 in CD4 T cells of RA were detected by Western blot. (I and J) CD4 T cells from RA patients were isolated and treated with HS and/or GSK for 3 days, and the proportion of Th17 cells was detected by flow cytometry. (K and L) CD4 T cells from RA patients were isolated and treated with HS and/or GSK for 3 days, and the proportion of Treg cells was detected by flow cytometry. Statistical significance was calculated by unpaired Student’s *t* test and data are represented as mean ± SD. The correlation was determined by linear regression analysis for (D) and (E). ∗*p* < 0.05, ∗∗*p* < 0.01, ∗∗∗*p* < 0.001, ns not significant.

To demonstrate the roles of PDPK1 and SGK1 in CD4 T cells of RA patients, peripheral CD4 T cells from RA patients and healthy controls (HCs) were isolated and subjected to western blot analysis. It was noted that the phosphorylated PDPK1 and SGK1 were higher in RA patients as compared to those of healthy individuals (Figures 1A–1C). Particularly, the phosphorylated PDPK1 (Figure 1D) and SGK1 (Figure 1E) levels exhibited a positive correlation with the DAS28-ESR score, an indicator of RA severity. We then decided to utilize PDPK1/SGK1 inhibitor GSK for the *in vitro* validation. At the concentration of 2.5 μmol/L, GSK displayed no observable impact on either CD4 T cell apoptosis (Figures S1A–S1C) or proliferation (Figure S1D). However, at the concentrations of 5 and 10 μmol/L, GSK notably augmented CD4 T cell apoptosis (Figures S1A–S1C) and hampered their proliferation (Figure S1D). Therefore, 2.5 μmol/L of GSK was applied in subsequent experiments. Specifically, exposure of RA derived CD4 T cells to HS further enhanced the phosphorylated levels of both PDPK1 and SGK1 (Figures 1F–1H), resulting in an increased Th17 proportion (Figures 1I and 1J) and a decline in Treg frequency (Figures 1K and 1L). In contrast, GSK treatment attenuated PDPK1 and SGK1 phosphorylation (Figures 1F–1H), by which it corrected the Th17 enhancement and Treg impediment triggered by HS treatment (Figures 1I–1L).

Next, we divided RA patients into low salt (≤5g/day) and HS (>5g/day) diet group based on their daily salt consumption. Clinical variable analysis unveiled that patients in the HS diet group exhibited elevated phosphorylated levels for PDPK1 and SGK1, along with a higher number of affected joints and increased DAS28-ESR scores (*p* < 0.05). In contrast, no significant difference was identified between the two dietary groups in terms of clinical indices, such as the ESR level, disease duration, rheumatoid factor (RF) positivity, anti-cyclic citrullinated peptide (anti-CCP) antibody positivity, gender, and age (Table 1). Overall, these data suggest a possible connection between HS, PDPK1/SGK1, and disease activity of RA patients.

**Table 1:** Comparison of clinical characteristics between RA patients on a low-salt diet versus RA patients on a high-salt diet

	Low-Salt Diet RA patients (*n* = 7)	High-Salt Diet RA patients (*n* = 12)	*p*-Value
*p*-SGK1 Relative Expression (mean ± SD)	0.54 ± 0.11	0.66 ± 0.10	0.020
*p*-PDPK1 Relative Expression (mean ± SD)	0.50 ± 0.06	0.57 ± 0.06	0.017
Number of Tender Joints (mean ± SD)	3.71 ± 1.11	6.17 ± 2.25	0.016
Number of Swollen Joints (mean ± SD)	1.71 ± 1.80	3.67 ± 2.50	0.089
ESR (mean ± SD)	31.3 ± 8.20	39.92 ± 10.31	0.076
DAS28-ESR (mean ± SD)	4.21 ± 0.59	5.04 ± 0.64	0.013
Disease Duration (months, mean ± SD)	6.00 ± 1.92	6.75 ± 2.01	0.435
RF Positive (%)	85.7	75.0	1.000
anti-CCP Antibody Positive (%)	100	75	0.263
Female (%)	71.4	83.3	0.603
Age (years, mean ± SD)	33.86 ± 5.70	33.17 ± 5.13	0.789

ESR: Erythrocyte Sedimentation Rate; DAS28-ESR: Disease Activity Score based on 28 Joints with ESR; RF: Rheumatoid Factor; anti-CCP Antibody: Anti-Cyclic Citrullinated Peptide Antibody. *p* < 0.05 indicates statistically significant difference.

### GSK reverses the effects of HS on Th17 and Treg program

**Figure 2:**
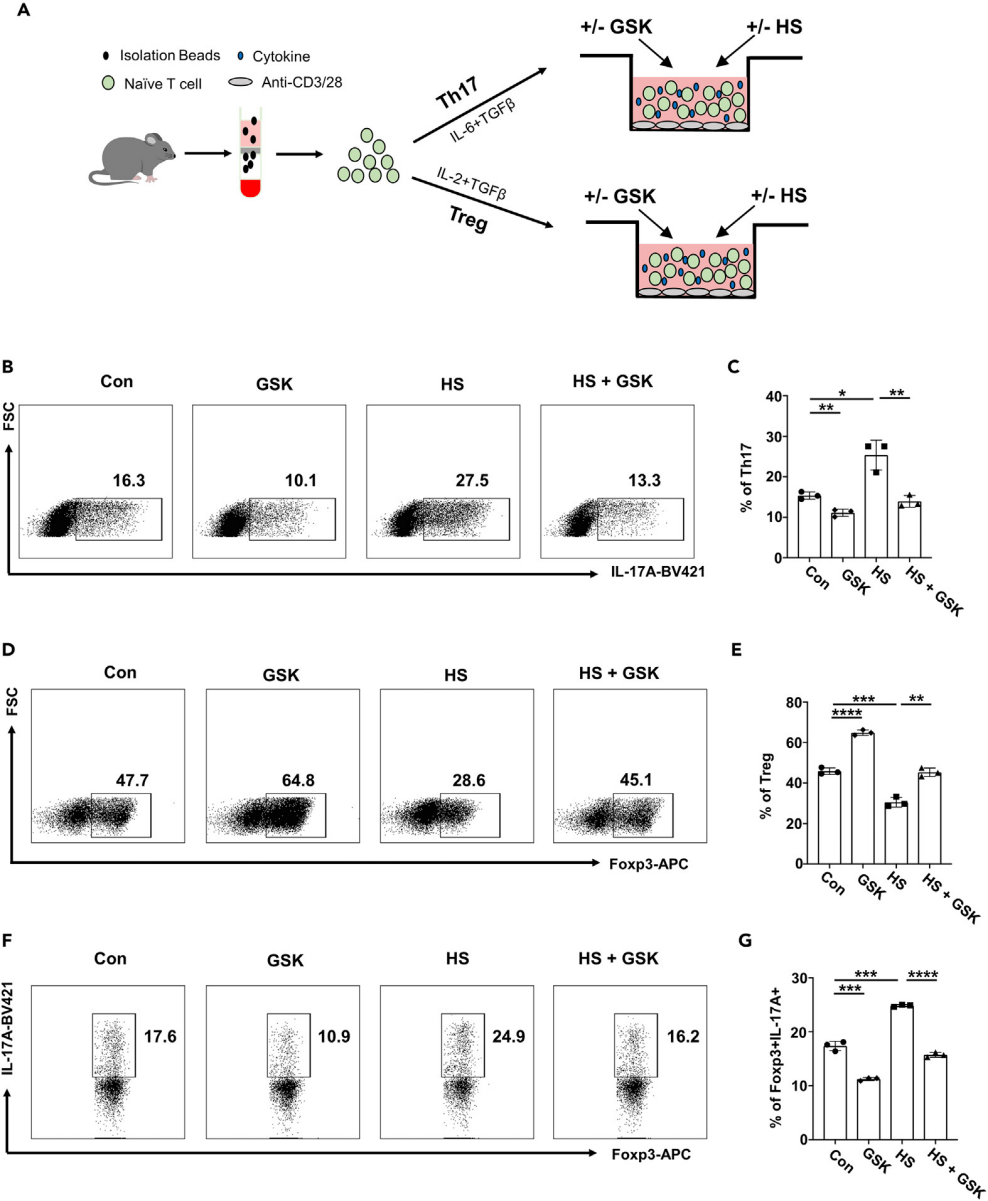
GSK reverses the effects of HS on Th17 and Treg program *in vitro* (A) Schematic representation of Th17 and Treg *in vitro* differentiation. (B and C) The proportion of differentiated Th17 cells (*n* = 3). (D and E) The proportion of differentiated Treg cells (*n* = 3). (F and G) Naive CD4 T cells were cultured for 3 days under the Treg polarization condition. Then, Treg cells were harvested, washed and cultured for an additional 2 days under Th17 condition in the presence of HS and/or GSK. The proportion of IL-17A producing cells within the Foxp3^+^ Treg cells was shown (*n* = 3). Statistical significance was calculated by unpaired Student’s *t* test and data are represented as mean ± SD. ∗*p* < 0.05, ∗∗*p* < 0.01, ∗∗∗*p* < 0.001, ∗∗∗∗*p* < 0.0001.

Based on the above findings, we next sought to address whether GSK affects the polarization of Th17 and Treg cells, particularly under a HS condition (Figure 2A). As expected, HS treatment markedly promoted Th17 differentiation, whereas GSK suppressed the Th17 program either used alone or in the context of HS treatment (Figures 2B and 2C). In contrast, HS substantially hindered the induction of Treg cells, which could be reversed by co-administration of GSK (Figures 2D and 2E). The Treg cells were next challenged under a Th17 polarizing condition. Strikingly, HS prominently bolstered the IL-17A levels in Foxp3^+^ Treg cells, while GSK prevented the ectopic expression of IL-17A with or without HS treatment (Figures 2F and 2G).

**Figure 3:**
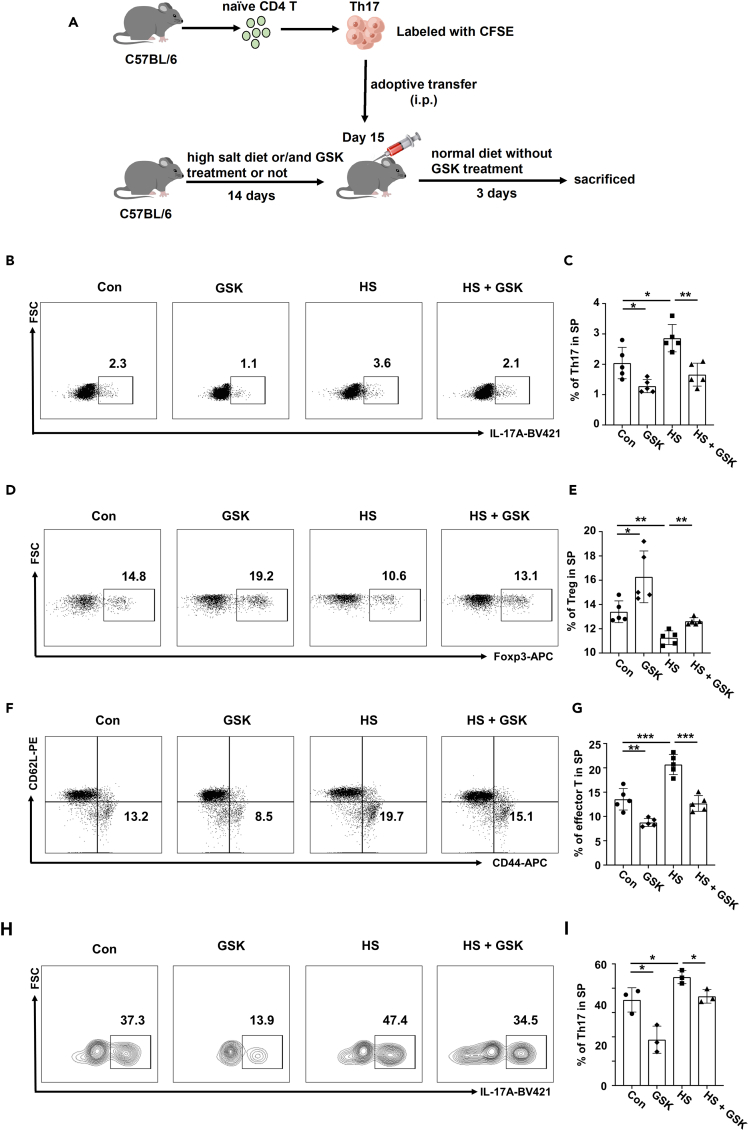
GSK reverses the effects of HS on Th17 and Treg program *in vivo* (A) Schematic representation of the adoptive transfer model. (B and C) The proportion of Th17 cells within splenic CD4 T cells of recipient mice (*n* = 5). (D and E) The proportion of Treg cells within splenic CD4 T cells of recipient mice (*n* = 5). (F and G) The proportion of activated CD4 T cells within splenic CD4 T cells of recipient mice (*n* = 5). (H and I) The proportion of IL-17A producing donor cells in spleen (*n* = 3). Statistical significance was calculated by unpaired Student’s *t* test and data are represented as mean ± SD. ∗*p* < 0.05, ∗∗*p* < 0.01, ∗∗∗*p* < 0.001.

In order to corroborate the *in vitro* observations, we adoptively transferred labeled Th17 cells into host mice pretreated with a control vehicle (Con), HS, GSK or HS + GSK, respectively (Figure 3A). Analysis of recipient-derived CD4 T cells revealed that mice in the HS group displayed an increased proportion of Th17 cells in both spleen (Figures 3B and 3C) and lymph nodes (Figure S2A) as compared to that of mice in the Con group, while mice in the GSK and HS + GSK groups were characterized by a lower Th17 frequency. Moreover, mice in the HS group exhibited a decreased proportion of Treg cells in the spleen (Figures 3D and 3E) and lymph nodes (Figure S2B), and the phenotypes could be partially rescued by GSK treatment. Consistently, in both spleen and lymph nodes, the Th17/Treg ratio within recipient-derived CD4 T cells increased in HS group, comparing to Con mice, and such increment could be abolished by GSK (Figures S2E and S2F). In line with these findings, the proportion of activated CD4 T cells was increased in the HS group but decreased in the GSK and HS + GSK groups in the spleen (Figures 3F and 3G) and lymph nodes (Figure S2C) as compared to that of mice in the control group. Similarly, in a separate analysis gated on donor cells, we found that the HS group had increased proportion of IL-17A producing cells in the spleen (Figures 3H and 3I) and lymph nodes (Figure S2D), while the GSK and HS + GSK groups showed a lower frequency.

**Figure 4:**
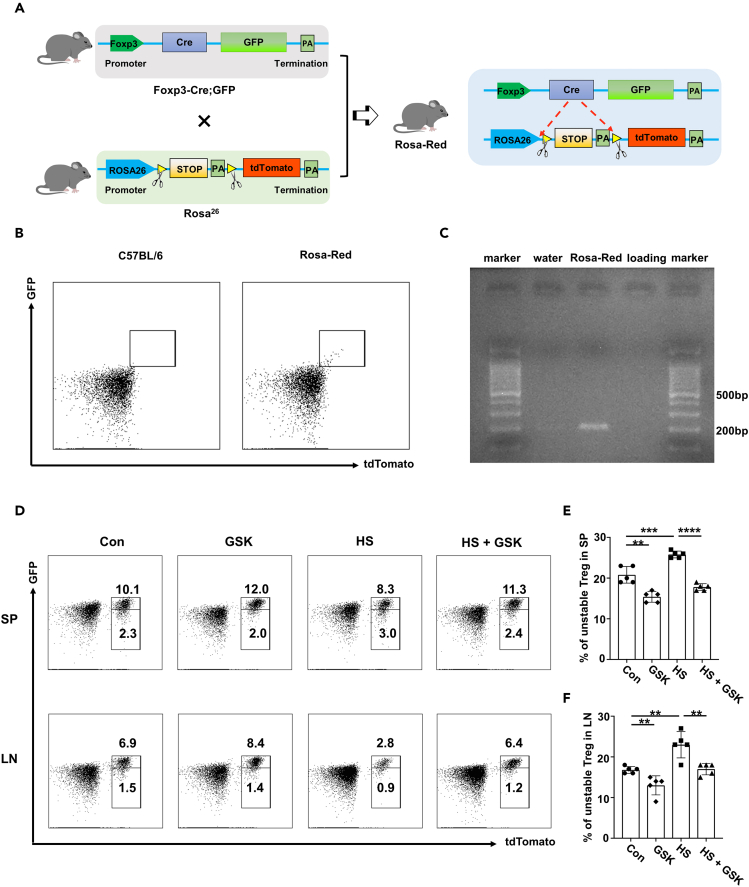
GSK rectifies high salt-induced Treg instability *in vivo* (A) Schematic representation of constructing Rosa-Red mouse model. (B) Peripheral one-drop blood flow cytometry was employed to validate Rosa-Red transgenic mice. (C) PCR was employed for genotyping Rosa-Red mice. (D–F) The proportion of unstable Treg cells (tdTomato^+^ GFP^−^) within the total Treg population in the spleen and lymph nodes of Rosa-Red mice. Statistical significance was calculated by unpaired Student’s *t* test and data are represented as mean ± SD. ∗∗*p* < 0.01, ∗∗∗*p* < 0.001, ∗∗∗∗*p* < 0.0001.

To further investigate the *in vivo* impact of HS and GSK on Treg cells, we generated the Treg lineage tracing mice (Foxp3^Cre-eGFP^; Rosa26^flox-stop-flox-tdTomato^, defined as Rosa-Red) as detailed in Figure 4A. The successful construction of Rosa-Red mice was confirmed by one-drop blood FACS staining (Figure 4B) and PCR genotyping (Figure 4C). Remarkably, the HS group displayed an elevated proportion of tdTomato^+^ GFP^−^ unstable Treg cells within the total Treg population as compared to the control group, whereas GSK treatment reduced the frequency of unstable Tregs (Figures 4D and 4E). Similarly, in the lymph nodes, the proportion of unstable Tregs within the CD4 T cell population was decreased in the HS group, which was rescued by the GSK treatment (Figures 4D and 4F). Taken together, our findings confirmed that GSK rectifies HS-triggered Th17/Treg imbalance through inhibiting Th17 differentiation, promoting the Treg program, and enhancing Treg stability.

### GSK ameliorates HS-exacerbated progression of arthritis

**Figure 5:**
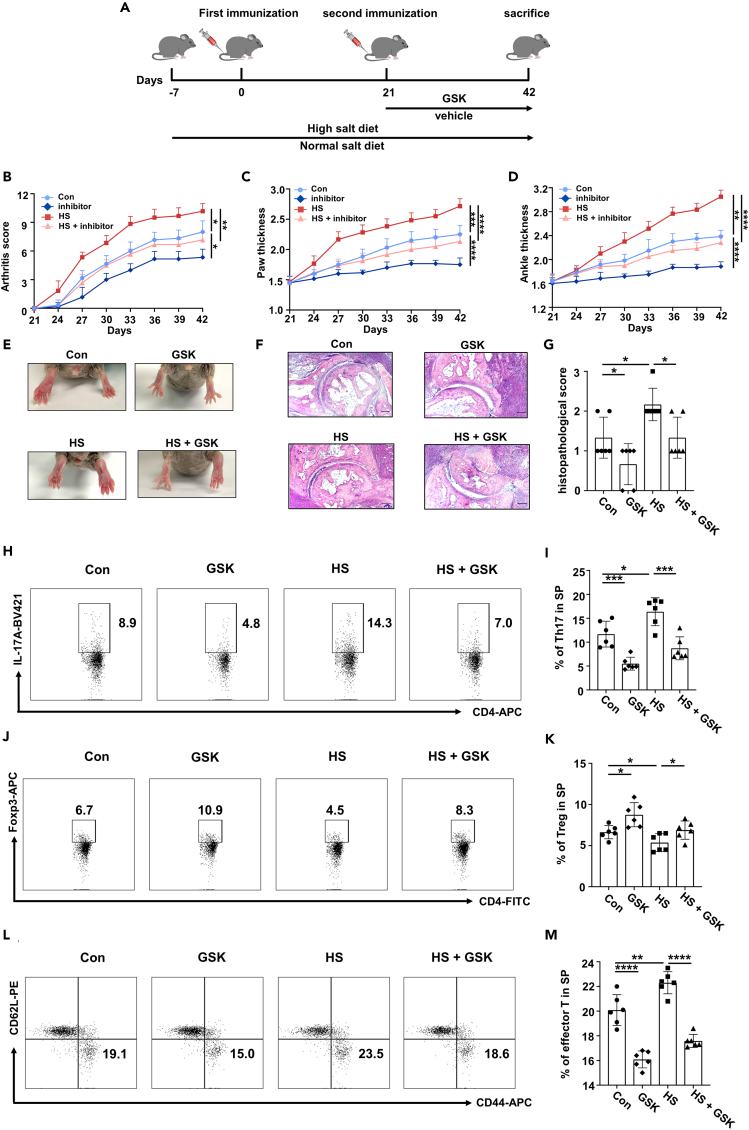
GSK ameliorates high salt-accelerated arthritis progression (A) Schematic representation of CIA model. (B) Arthritis score of CIA mice (*n* = 6). (C) Paw thickness of CIA mice (*n* = 6). (D) Ankle thickness of CIA mice (*n* = 6). (E) Representative pictures of the hind paw joints of CIA mice at day 42. (F) H&E staining of ankle joints of CIA mice. Scale bars, 100 μm. (G) The histopathological score of knee joints of CIA mice. (H and I) The proportion of Th17 cells within splenic CD4 T cells of CIA mice (*n* = 6). (J and K) The proportion of Treg cells within splenic CD4 T cells of CIA mice (*n* = 6). (L and M) The proportion of activated CD4 T cells within splenic CD4 T cells of CIA mice (*n* = 6). Statistical significance was calculated by unpaired Student’s *t* test and data are represented as mean ± SD. ∗*p* < 0.05, ∗∗*p* < 0.01, ∗∗∗*p* < 0.001, ∗∗∗∗*p* < 0.0001.

To assess the effects of HS and GSK on RA progression, we employed a CIA model in RA-susceptible DBA/1 mice. DBA/1 mice were fed with either a normal diet or a HS diet prior to the first immunization. Treatment with either GSK (25 mg/kg, every three days) or a control vehicle began immediately after the second immunization (Figure 5A). Our results showed that the HS diet significantly intensified arthritic symptoms, as evidenced by the elevated arthritis scores (Figure 5B), increased paw and ankle thickness (Figures 5C and 5D), and worsened joint pathology (Figures 5E–5G). Conversely, GSK treatment alleviated these arthritic symptoms and counteracted the adverse effects of HS diet on RA progression (Figures 5B–5G). In line with these observations, GSK-treated CIA mice displayed a reduced proportion of Th17 cells (Figures 5H, 5I, S3， andS4A) along with an increased proportion of Treg cells (Figures 5J, 5K; S4B) in the spleen and lymph nodes. In contrast, mice on HS diet exhibited an elevated frequency of Th17 cells and a reduced proportion of Treg cells that were efficiently reversed by GSK treatment in the spleen (Figures 5H–5K) and lymph nodes (Figures S4A and S4B). Consistently, in both spleen and lymph nodes, the Th17/Treg ratio increased in CIA mice fed with a high-salt diet when compared to Con mice, while GSK treatment could reverse such effect (Figures S4G and S4H). Similarly, the percentages of activated CD4 T cells both in the spleen (Figures 5L and 5M) and lymph nodes (Figure S4C) were significantly higher in the HS group, while GSK treatment efficiently dampened T cell activation.

Given that the Th17 and Treg frequencies were altered following treatment with GSK or HS, we then evaluated the serum levels of cytokines associated with these two T cell subsets. Strikingly, compared to control mice, pro-inflammatory cytokines TNF-α and IL-6, were reduced in the GSK group but elevated in the HS group. In contrast, the anti-inflammatory cytokine IL-10 was higher in the GSK group and lower in the HS group. Moreover, compared to the HS group, co-administration of GSK and HS resulted in the down-regulation of TNF-α and IL-6 but up-regulation of IL-10 (Figures S4D–S4F). Altogether, our results support that GSK serves as a promising candidate for RA treatment, and the therapeutic effect is achieved through mitigating the Th17/Treg imbalance, especially in the case of consumption of a HS diet.

### GSK represses the glycolytic capacity of HS-insulted CD4 T cells

**Figure 6:**
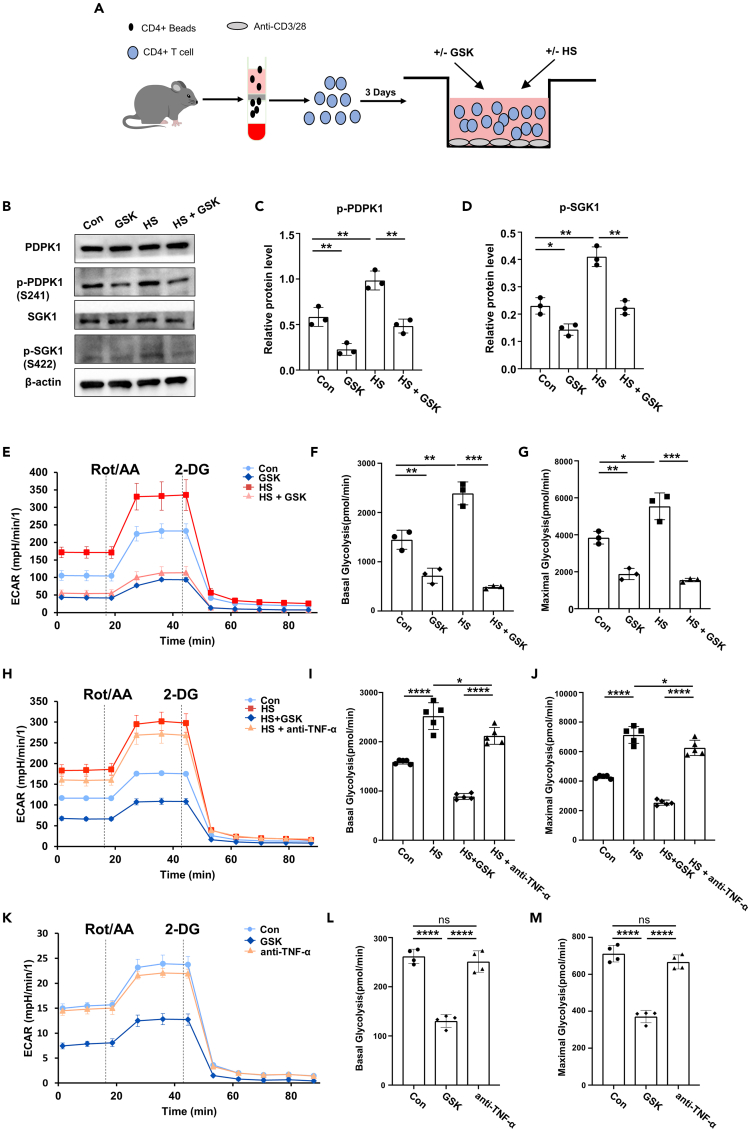
GSK down-tunes the glycolytic metabolism of high salt-insulted CD4 T cells (A) Schematic representation of CD4 T cell culture. (B) Expression levels of (p-)PDPK1 and (p-)SGK1 in CD4 T cells following treatment with GSK and/or HS (*n* = 3) were detected by western blot. (C and D) Quantitative analysis of *p*-PDPK1 and *p*-SGK1 expression (*n* = 3). (E) The glycolytic activity of CD4 T cells following GSK and/or HS treatment (*n* = 3). (F) Basal glycolysis rates post-treatment (*n* = 3). (G) Maximal glycolysis capacity of CD4 T cells following GSK and/or HS treatment (*n* = 3). (H) The glycolytic activity of CD4 T cells following co-treatment with HS and GSK or anti-TNF-α (*n* = 3). (I) Basal glycolysis rates of CD4 T cells following co-treatment with HS and GSK or anti-TNF-α (*n* = 3). (J) Maximal glycolysis rates following co-treatment with HS and GSK or anti-TNF-α (*n* = 3). (K) The glycolytic activity of CD4 T cells following treatment with PBS, GSK or anti-TNF-α (*n* = 3). (L) Basal glycolysis rates of CD4 T cells following treatment with PBS, GSK or anti-TNF-α (*n* = 3). (M) Maximal glycolysis rates following treatment with PBS, GSK or anti-TNF-α (*n* = 3). Statistical significance was calculated by unpaired Student’s *t* test and data are represented as mean ± SD. ∗*p* < 0.05, ∗∗*p* < 0.01, ∗∗∗*p* < 0.001, ∗∗∗∗*p* < 0.0001, ns (not significant).

To elucidate the mechanisms by which HS and GSK influence the Th17/Treg balance, we carried out the following experiments using splenic CD4 T cells isolated from C57BL/6 mice (Figure 6A). Upon activation with plate-coated anti-CD3 and anti-CD28 antibodies (TCR stimulation), CD4 T cells were treated with HS, GSK or HS + GSK for 3 days. GSK significantly repressed the phosphorylation of PDPK1 and SGK1, while the opposite results were noted in HS-treated cells (Figures 6B–6D). As PDPK1 and SGK1 are pivotally engaged in the regulation of glycolytic process, we conducted glucose uptake assay and found GSK prominently counteracted the enhancing effect of HS (Figures S5A and S5B). Seahorse metabolic analysis further confirmed that GSK diminished both basal and maximal glycolytic rates that were boosted by HS (Figures 6E–6G). Next, CD4 T cells from RA patients were treated with HS, GSK, HS + GSK, and HS + anti-TNF-α for 3 days followed by Seahorse analysis. Of note, GSK similarly reversed the glycolysis-promoting function of HS while anti-TNFα only demonstrated a limited effect (Figures 6H–6J). Further, CD4 T cells from RA patients were cultured under conditions without high-salt stimulation and treated with either PBS, GSK or anti-TNF-α for 3 days, followed by Seahorse analysis. Remarkably, GSK reduced both the basal and maximal glycolytic rates of CD4 T cells, whereas anti-TNF-α displayed no perceptible impact on CD4 T cell glycolysis when compared to the PBS group (Figures 6K–6M). Collectively, these data indicate that HS challenge promotes the glycolytic capacity of CD4 T cells by activating the PDPK1-SGK1 signaling, which could be reversed by the addition of GSK.

### Blockade of FoxO1 impairs the immune regulatory effect of GSK

**Figure 7:**
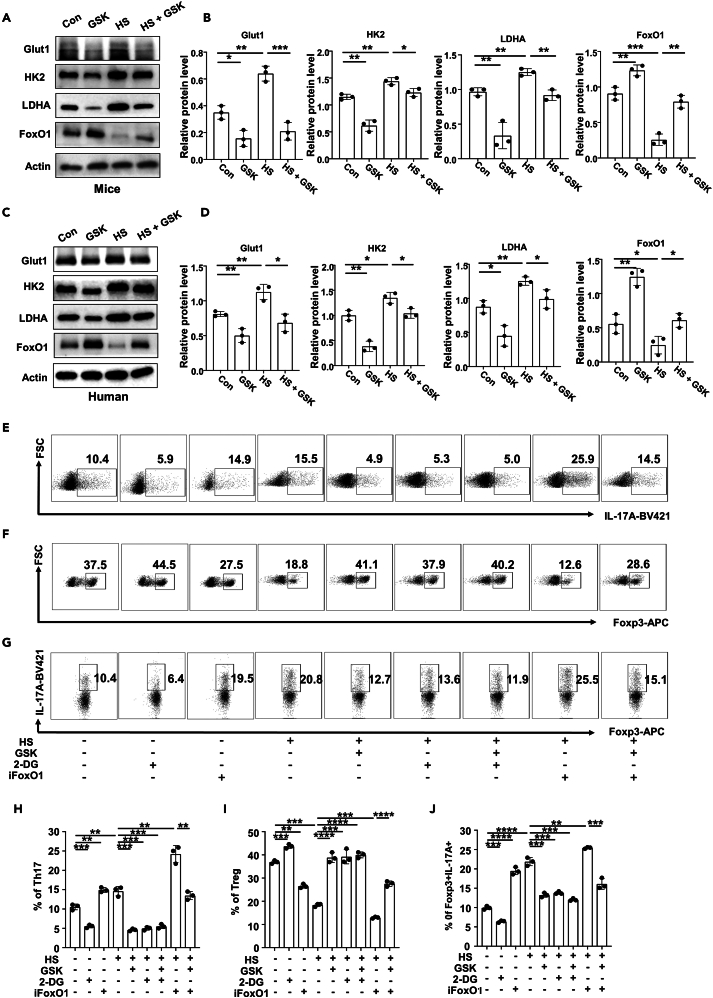
Blockade of FoxO1 impairs the immune regulatory effects of GSK (A and B) CD4 T cells were isolated from C57BL/6 mice and stimulated by anti-CD3/28 in the presence of GSK and/or HS or not for 3 days (A) Western blot analysis was employed to assess the expression levels of Glut1, HK2, LDHA, and FoxO1 in CD4 T cells from C57BL/6 mice (*n* = 3). (B) Quantitative analysis of Glut1, HK2, LDHA, and FoxO1 expression in CD4 T cells from C57BL/6 mice (*n* = 3). (C, D) CD4 T cells were isolated from peripheral blood of RA patients and stimulated by anti-CD3/28 in the presence of GSK and/or HS for 3 days. (C) Western blot analysis was employed to assess the expression levels of Glut1, HK2, LDHA, and FoxO1 in CD4 T cells from RA patients (*n* = 3). (D) Quantitative analysis of Glut1, HK2, LDHA, and FoxO1 expression in CD4 T cells from RA patients (*n* = 3). (E–J) Naive CD4 T cells from C57BL/6 mice were polarized into Th17 cells or Treg cells in the presence of different combinations of HS, GSK, 2-DG and iFoxO1 for 3 days. For Treg stability assay, naive CD4 T cells were initially cultured for 3 days under Treg polarization condition. Then, Treg cells were harvested, washed and cultured for an additional 2 days under Th17 condition in the presence of HS, GSK, 2-DG or iFoxO1. (E, H) The proportion of Th17 cells within CD4 T cells following distinct stimulation (*n* = 3). (F, I) The proportion of Treg cells within CD4 T cells following distinct stimulation (*n* = 3). (G, J) The proportion of IL-17A producing cells within total Foxp3^+^ Treg cells following distinct stimulation (*n* = 3). Statistical significance was calculated by unpaired Student’s *t* test and data are represented as mean ± SD. ∗*p* < 0.05, ∗∗*p* < 0.01, ∗∗∗*p* < 0.001, ∗∗∗∗*p* < 0.0001.

The above findings prompted us to check the expression of key molecules relevant to glycolysis and glucose uptake. Indeed, an HS insult induced the expression of HK2, LDHA and Glut1 both in mouse (Figures 7A and 7B) and human CD4 T cells (Figures 7C and 7D), which was reversed by the addition of GSK. It was interestingly noted that HS attenuated the expression of FoxO1, a crucial molecule downstream of SGK1 signaling, while its expression was restored upon the addition of GSK (Figures 7A–7D).

Given the role of FoxO1 played in GSK-mediated transcription of glycolytic effectors and IL-17A, we then embarked on the dependence of GSK on FoxO1-mediated glycolytic rewiring. To this end, CD4 T cells isolated from C57BL/6 mice were treated with FoxO1 inhibitor (iFoxO1) for 3 days. As expected, iFoxO1 significantly up-regulated the expression levels of Glut1, LDHA and HK2 (Figures S5C–S5F). We then polarized naive T cells to Th17 and Treg subsets under different combinations of HS, GSK, iFoxO1 and 2-DG (glycolysis inhibitor). Compared to the PBS control, the proportion of *in vitro* polarized Th17 cells increased upon HS treatment (Figures 7E and 7H). As both 2-DG and GSK inhibited Th17 program in the presence or absence of HS, iFoxO1 efficiently abolished the inhibitory effect of GSK (Figures 7E and 7H). Conversely, in respect to Treg induction, the proportion of *in vitro* polarized Treg cells decreased upon HS treatment (Figures 7F and 7I). However, 2-DG and GSK both restored the defective Treg program induced by HS, while iFoxO1 treatment diminished the rescuing effect of GSK (Figures 7F and 7I). We then checked Treg stability by probing the expression level of ectopic IL-17A. Compared to the PBS control, the expression levels of IL-17A in Treg cells elevated in the HS group (Figures 7G and 7J). While 2-DG and GSK restrained IL-17A ectopic expression in the presence or absence of HS, iFoxO1 treatment nullified the protective role of GSK (Figures 7G and 7J). Collectively, those data supported that GSK favorably rectifies the HS-triggered Th17/Treg imbalance, which depends on FoxO1-imposed restriction of glycolytic capacity and IL-17A production in CD4 T cells.

## Discussion

**Figure 8:**
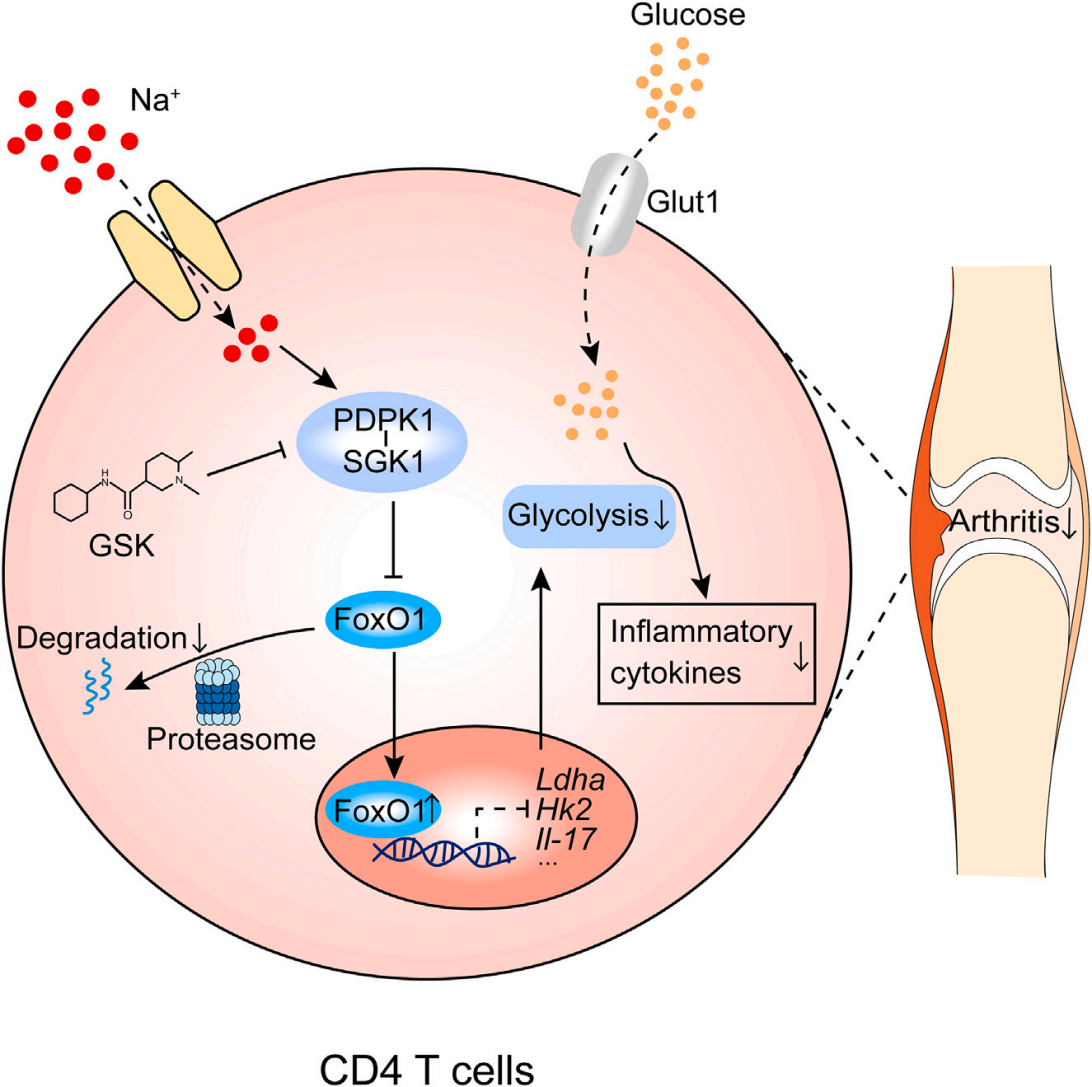
Graphical illustration High salt stimulation turbocharges the glycolytic metabolism of CD4 T cells through the PDPK1-SGK1-FoxO1 signaling pathway, which subsequently disturbs the Th17/Treg balance and promotes the progression of RA. Utilization of PDPK1/SGK1 inhibitor, GSK, effectively corrects Th17/Treg imbalance to mitigate the detrimental outcomes of high salt insult.

It was noted that mice fed a HS diet displayed worsened disease symptoms and elevated frequency of Th17 cells in the spleen of an EAE model.^43^ However, HS did not seemingly affect the quantity and function of dendritic cells (DCs), highlighting a direct impact of HS on the Th17 program. Herein in this report, we demonstrated evidence indicating that an HS insult significantly enhances the glycolytic capacity of CD4 T cells through the PDPK1-SGK1-FoxO1 axis, which subsequently disturbs the Th17/Treg program and promotes RA progression. Therefore, GSK, a PDPK1/SGK1 dual inhibitor, possesses the potency to effectively correct the Th17/Treg imbalance, thereby mitigating the detrimental outcomes resulting from an HS insult (Figure 8). We also provided preliminary clinical evidence that the elevated PDPK1/SGK1 phosphorylation level in CD4 T cells is highly correlated with daily salt intake and RA severity. It is noteworthy that our study only included a limited number of samples, follow-up studies with a larger RA cohort would be necessary.

Cellular metabolism is intricately connected to the functional adaptation of immune cells.^44^ Th17 cells predominantly rely on glycolysis for energy production, while Treg cells depend on mitochondrial metabolism and fatty acid oxidation for energy supply. The glycolytic capacity of Treg cells is significantly lower than that of Th17 cells.^45^ We now demonstrated that an HS insult promotes glycolytic capacity to impede Treg program. FoxO1, negatively regulated by SGK1, plays a dual role in Th17 and Treg subsets.^46^ On the one hand, FoxO1 promotes the expression of Foxp3 and other signature genes essential to Treg function.^47^^,^^48^ On the other hand, FoxO1 overexpression suppresses glycolytic capacity coupled with an attenuated Th17 program.^49^^,^^50^^,^^51^ Strikingly, extracellular HS environment elevates the intracellular Na^+^ level and disrupts Treg mitochondrial respiration through interfering with the electron transport chain (ETC), thereby impairing Foxp3 expression and enabling the ectopic expression of pro-inflammatory mediators.^52^ Furthermore, oxidative stress could be potently elicited by HS intake,^53^ and it is much likely that the metabolic rewiring and stress response are intertwined to mediate the pathologic consequences of HS stimulation.

Immune therapeutics have proven their value in RA treatment, especially for the currently developed biological agents.^54^ Although biologics such as IL-6 blockers and TNF-α neutralizing antibodies manifested notable efficacy in RA, a considerable population of patients, however, showed a mere suboptimal response.^55^ The insufficient responsiveness is ascribed to various factors,^56^ while the roles of an unhealthy lifestyle and bad dietary habits could not be excluded.^57^ Our study indicated that inhibition of PDPK1/SGK1 signaling by GSK significantly reduced the proportion of Th17 cells in HS-fed CIA mice, which was coupled with a marked decrease of circulating IL-6 and TNF-α levels, an increased proportion of Treg cells along with an enhanced IL-10 secretion. These findings support that targeting PDPK1/SGK1 signaling by GSK could be a viable strategy against RA in clinical settings.

### Limitations of the study

Our study has several limitations. First, due to difficulties in obtaining specimens, we did not assess the expression levels and phosphorylation states of PDPK1 and SGK1 in synovial tissue of RA patients. Consequently, experiments with humanized animal model were not conducted. At last, though we preliminarily revealed the association between salt intake and RA severity, large-population based epidemiological survey and prospective cohort studies are demanded to verify the causal relationship by checking more immunological parameters.

## STAR★Methods

### Key resources table

**Table:** 

REAGENT or RESOURCE	SOURCE	IDENTIFIER
**Antibodies**		

Anti-human CD3	Biolegend	Cat#300302; RRID:AB_314037
Anti-human CD28	Biolegend	Cat#302902; RRID:AB_314303
Anti-mouse CD3	Biolegend	Cat#100302; RRID:AB_312666
Anti-mouse CD28	Biolegend	Cat#102102; RRID:AB_312866
FITC anti-mouse CD4	Biolegend	Cat#100406; RRID:AB_312690
Brilliant Violet 421™ anti-mouse IL-17A	Biolegend	Cat#506926; RRID:AB_10900442
Alexa Fluor 647 anti-mouse Foxp3	Biolegend	Cat#126408; RRID:AB_1089115
PE anti-mouse CD44	Biolegend	Cat#103008; RRID:AB_312958
APC anti-mouse CD62L	Biolegend	Cat#104412; RRID:AB_313099
PE/Cy7 anti-mouse CD25	Biolegend	Cat#101916; RRID:AB_2616762
Alexa Fluor 647 anti-human Foxp3	Biolegend	Cat#320211; RRID:AB_430886
PE anti-human IL-17A	Biolegend	Cat#512306; RRID:AB_961395
Anti-PDPK1 (phospho S241)	Abcam	Cat#ab131098; RRID:AB_11159760
Anti-HK2	Abcam	Cat#ab209847; RRID:AB_2904621
Anti-LDHA	Abcam	Cat#ab52488; RRID:AB_2134961
Anti-PDPK1	Santa Cruz Biotechnology	Cat#sc-17765; RRID:AB_626657
Anti-β-Actin	Santa Cruz Biotechnology	Cat#sc-47778; RRID:AB_626632
Anti-SGK1	ABclonal	Cat#A1025; RRID:AB_2757776
Anti-SGK1 (phospho S422)	Absin	Cat#abs147953; N.A
Anti-Glut1	Proteintech	Cat#21829-1-AP; RRID:AB_10837075
Anti-FoxO1	proteintech	Cat#18592-1-AP; RRID:AB_10860103

**Biological samples**		

RA patients' peripheral blood samples	Tongji Hospital	N/A
Healthy Controls’ peripheral blood samples	Tongji Hospital	N/A

**Chemicals, peptides, and recombinant proteins**		

GSK2334470 (GSK)	MedChemExpress	HY-14981
Protease inhibitor cocktail	Roche	4693116001
RIPA lysis buffer	Beyotime	P0013K
Recombinant murine IL-2	Biolegend	#575402
Recombinant murine IL-6	Biolegend	#575702
Recombinant murine TGF-β	Biolegend	#763102
Recombinant human IL-2	Biolegend	#791906
Recombinant human IL-6	Biolegend	#570806
Recombinant human TGF-β	Biolegend	#583301
Anti-TNF-α (Etanercept)	MedChemExpress	HY-108847

**Critical commercial assays**		

Mouse inflammation kit	BD Biosciences	552364
MojoSort™ Mouse CD4 Naive T cell Isolation Kit	Miltenyi Biotec	130-104-453
Annexin V-FITC/PI Apoptosis Detection Kit	Yeasen Biotechnology	40302
ECL kit	Biosharp Life Sciences	BL520A
CFSE	Thermo Fisher	C34554

**Experimental models: Organisms/strains**		

DBA/1 mice	Jackson Laboratory	N/A
C57BL/6 mice	Jackson Laboratory	N/A
Foxp3Cre-eGFP; Rosa26flox-stop-flox-tdTomato mice (Rosa-Red mice)	Jackson Laboratory	N/A

**Software and algorithms**		

FlowJo software (v10.5.3)	BD Biosciences	https://www.bdbiosciences.com
GraphPad Prism 5.0 software	GraphPad Software Inc.	https://www.graphpad.com/; RRID:SCR_002798
FCAP ArrayTM Software	BD Biosciences	https://www.bdbiosciences.com

### Resource availability

#### Lead contact

Further information and requests for resources and reagents should be directed to and will be fulfilled by the lead contact, Cong-Yi Wang (wangcy@tjh.tjmu.edu.cn).

#### Materials availability

This study did not generate new unique reagents.

#### Data and code availability

• All data reported in this paper will be shared by the lead contact upon request.
• This paper does not report original code.
• Any additional information required to reanalyze the data reported in this paper is available from the lead contact upon request.

### Experimental model and subject details

#### Human subjects

In the study, fresh peripheral blood samples were collected from treatment-naïve patients who were newly diagnosed with RA (*n* = 19) according to the 2010 American College of Rheumatology/European League Against Rheumatism (ACR/EULAR) classification criteria for RA. Among the included RA patients, there were 15 females and 4 males. Healthy controls (*n* = 16, 13 females and 3 males) included in this study were matched for gender and age with RA patients. The average age of RA patients was 33 years, while the average age of HC individuals was 34 years. All participants are of Asian descent. Data collected from RA patients included age, gender, disease duration, daily salt intake, the number of tender joints in the 28 joints (TJC28), the number of swollen joints in the 28 joints (SJC28), the status of anti-CCP antibodies and RF, serum level of ESR and disease activity (Disease Activity Score-28 based ESR, DAS28-ESR). This study was approved by the Ethics Committee of Tongji Hospital, Tongji Medical College of Huazhong University of Science & Technology (project identification code: TJ -IRB20220973).

#### Experimental animal models

Collagen-induced arthritis (CIA) mouse model was established in male DBA/1 mice by injecting them with bovine type II collagen emulsified in complete Freund’s adjuvant, followed by a boost 21 days later with type II collagen emulsified in incomplete Freund’s adjuvant. The development of arthritis was monitored and arthritis scores were measured every 2 days. The Level of inflammation in each paw was graded on a scale from 0 to 4 as follows: 0, no inflammation; 1, detectable swelling in a single digit; 2, paw with swelling in more than one digit; 3, paw with swelling of all digits and instep; 4, severe swelling of the paw and ankle. The arthritis scores were the sum of the scores for all four paws. For the CIA model, mice on a high salt diet were fed sodium-rich chow containing 4% NaCl and provided with water containing 1% NaCl *ad libitum* from 7 days before the first immunization to 42 days thereafter. GSK2334470 (GSK) (25 mg/kg, every three days) or a vehicle control was administered to the mice via intraperitoneal injection from day 21 to day 42 following the second immunization. The vehicle for GSK consisted of 5% DMSO, 40% PEG300, 5% Tween-80 and 50% saline. On day 42 post-first immunization, blood samples, ankle joints, spleen and mesenteric lymph node (MLN) were collected from the mice. In the Adoptive transfer model C57BL/6 mice (6–8 weeks old) were fed with a standard chow diet and provided with water *ad libitum*, or they were fed a high salt diet (sodium-rich chow containing 4% NaCl and water containing 1% NaCl *ad libitum*) from day 0 to day 14. Concurrently, the mice were subjected to intraperitoneal injections of GSK (25 mg/kg, every three days) or vehicle control. Naive CD4 T cells from untreated C57BL/6 mice (8 weeks old) were polarized into Th17 cells in the presence of IL-6 (50 ng/mL) and TGF-β (2.5 ng/mL).^58^ The differentiated Th17 cells were then labeled with CFSE and transferred into the recipient mice that had been pretreated with a high salt diet and/or GSK via intraperitoneal injection upon the last dose of GSK administration (day 15). Spleens and MLNs were collected on day 18 for immunophenotyping.

We generated the Treg lineage tracing mice (Foxp3^Cre-eGFP^; Rosa26^flox-stop-flox-tdTomato^, defined as Rosa-Red) by crossing Foxp3^Cre-eGFP^ mice with Rosa26^flox-stop-flox-tdTomato^ mice. Treg lineage tracing Rosa-Red mice (6–8 weeks old) were either fed a standard chow diet and provided with water *ad libitum*, or placed on a high salt diet consisting of sodium-rich chow containing 4% NaCl and water with 1% NaCl *ad libitum*, and were given intraperitoneal injections of GSK (25 mg/kg, three times a week) or vehicle for 14 days. The spleen and MLN were collected on day 14 for flow cytometry analysis.

All animal experiments were conducted in accordance with the National Institutes of Health guidelines for the care and use of laboratory animals and were approved by the Tongji Hospital Animal Care and Use Committee (project identification code: TJH-202206029).

### Method details

#### Cell culture and polarization

For Th17 or Treg polarization, naive CD4 T cells were isolated from the spleens of C57BL/6 mice using a mouse naive CD4 T cell isolation kit (Miltenyi Biotec, 130-104-453). Naive CD4 T cells were cultured for 3 days with anti-CD3 and anti-CD28 stimulation either in Th17 polarization condition (IL-6 50 ng/mL, TGF-β 2.5 ng/mL) or Treg polarization condition (IL-2 10 ng/mL, TGF-β 5 ng/mL). For Treg stability assay, naive CD4 T cells were initially cultured for 3 days with the stimulation of anti-CD3 and anti-CD28 under Treg polarization condition, and the differentiated Treg cells were harvested, washed and cultured for an additional 2 days under Th17 condition. In some wells, the indicated stimulant(s) and chemical compound(s) were added in the final concentrations of 40 mmol/L HS,^6^ 2.5 μmol/L GSK, 1 mmol/L 2-DG, and 25 nmol/L iFoxO1.

#### T cell proliferation and apoptosis assay

Naive CD4 T cells from spleens of C57BL/6 mice were labeled with CFSE (Life Technologies, Carlsbad, CA) and cultured for 3–5 days under the stimulation of plate-coated anti-CD3 and anti-CD28. The mean fluorescence intensity (MFI) of CFSE was analyzed by flow cytometry. Similarly, naive CD4 T cells from spleens of C57BL/6 mice were isolated and cultured for 24 h for the T cell apoptosis assay. The apoptotic ratio was measured by the Annexin V-FITC/PI staining, employing the Annexin V-FITC/PI Apoptosis Detection Kit (Yeasen, 40302) according to the manufacturer’s instructions.

#### Western blot analysis

Western blot was conducted as previously reported.^59^ Cells were isolated and lysed with cold RIPA lysis buffer from Beyotime (Shanghai, China), which was enriched with a protease inhibitor cocktail from Roche (IN, USA). Proteins were separated on SDS-PAGE gels and subsequently transferred to PVDF membranes. These membranes were then probed with primary antibodies. β-actin was served as the loading control to ensure consistent protein loading across the samples. Following the primary antibody incubation, the membranes were exposed to the corresponding HRP-conjugated secondary antibodies. For visualization, the membranes were treated using an ECL kit (Biosharp Life Sciences, BL520A). The protein bands were viewed and analyzed for relative intensities using the GelView 6000Plus Smart Chemiluminescence Imaging System.

#### Flow cytometry analysis

A single-cell suspension from spleen or lymph nodes was prepared. For cell surface staining, cells were incubated with specified antibodies and incubated for 30 min on ice. Subsequent intracellular staining was performed using the Transcription Factor Buffer Set alongside the selected antibody cocktails. To identify intracellular cytokines, cells were stimulated with the Cell Activation Cocktail for a duration of 4–6 h. The antibodies used included FITC anti-mouse CD4 (#100406), APC anti-mouse CD62L (#104412), PE/Cy7 anti-mouse CD25 (#101916), Alexa Fluor 647 anti-mouse Foxp3 (#126408), Brilliant Violet 421 anti-mouse IL-17A (#506926), and PE anti-mouse CD44 (#103008). All antibodies were derived from Biolegend (San Diego, CA, USA). A consistent antibody dilution of 1/200 was used to ensure optimal results. After the staining procedure, the data were acquired with the MACSQuant Analyzer 10 from Miltenyi Biotec and processed using the FlowJo software (v10.5.3). In this study, CD4^+^ IL-17A^+^ cells are denoted as Th17 ^60^ and CD4^+^ Foxp3^+^ cells are denoted as Treg.^61^

#### Measurement of serum cytokines

Serum cytokines were measured using the mouse inflammation kits (BD Biosciences, 552364) according to the manufacturer’s instructions. Briefly, the capture beads, samples and PE-conjugated detection antibodies were incubated together at room temperature for 2 h. Data were collected with flow cytometry, and the results were generated using FCAP Array Software.

### Quantification and statistical analysis

The correlation was determined by linear regression analysis. All other data are expressed as mean ± SD. Student’s t test was applied for comparisons between two groups. Once more than two groups were compared, one-way or two-way analysis of variance was employed. In all cases, *p* < 0.05 was considered as statistically significant. Statistical analyses were conducted using the GraphPad Prism 5.0 software (GraphPad Software Inc., San Diego, CA, USA).

#### Study approval

All animal care and experimental procedures were approved by the Animal Care and Use Committee of Tongji Hospital, Tongji Medical College, Huazhong University of Science and Technology (TJH-202206029), and conducted in accordance with NIH guidelines. The studies in human samples were approved by the Ethics Committee of Tongji Hospital, Tongji Medical College of Huazhong University of Science and Technology (TJ-IRB20220973).
