# Supplementary material for: GSK2334470 attenuates high salt-exacerbated rheumatoid arthritis progression by restoring Th17/Treg homeostasis
Source: iScience. 2024 Apr 26;27(6):109798. doi: 10.1016/j.isci.2024.109798 (PMC11214488; doi:10.1016/j.isci.2024.109798)
Supplement: Document S1. Figures S1–S5 [file mmc1.pdf]

iScience, Volume *27*

Supplemental information

GSK2334470 attenuates high salt-exacerbated rheumatoid arthritis progression by restoring Th17/Treg homeostasis

Qian Mo, Mansoor Bolideei, Shan-Jie Rong, Jia-Hui Luo, Chun-Liang Yang, Wan-Ying Lu, Qi-Jie Chen, Jia-Wei Zhao, Fa-Xi Wang, Ting Wang, Yang Li, Xi Luo, Shu Zhang, Fei Xiong, Qi-Lin Yu, Zi-Yun Zhang, Shi-Wei Liu, Fei Sun, Ling-Li Dong, and Cong-Yi Wang

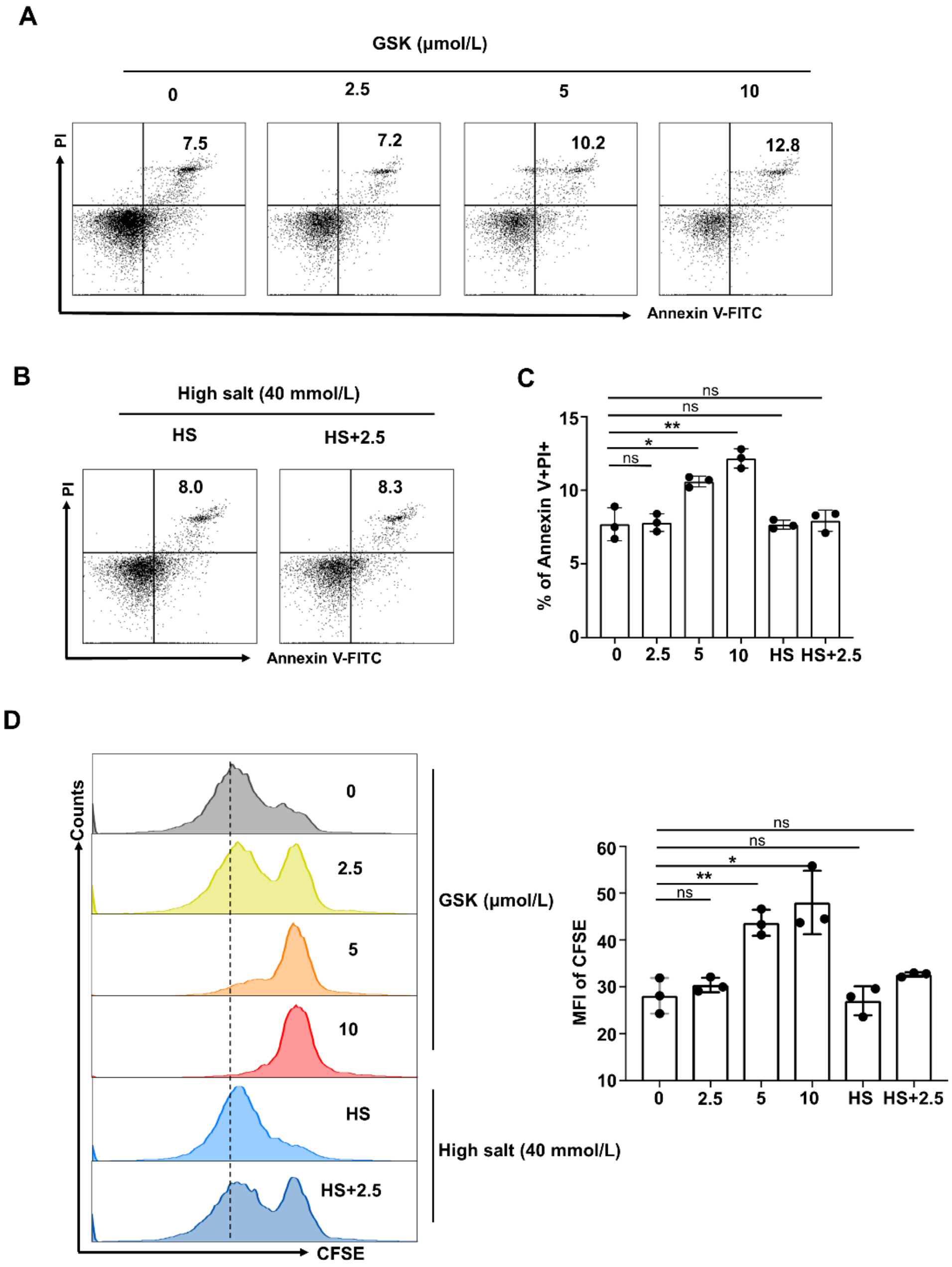

Figure S1. [The impact of GSK and HS on CD4 T cell proliferation and apoptosis], Related to Figure 2. (A, B, C) The effects of HS and varying concentrations of GSK on CD4 T cell apoptosis. No significant alteration in cell apoptosis was observed when GSK was applied at a concentration of 2.5 μmol/L (n = 3). **(D)** The effects of HS and varying concentrations of GSK on CD4 T cell proliferation. A slight alteration in cell proliferation was observed at a concentration of 2.5 μmol/L (n = 3). Statistical significance was calculated by unpaired Student's *t* test and data are represented as mean ± SD. *p<0.05, **p<0.01, ns (not significant).

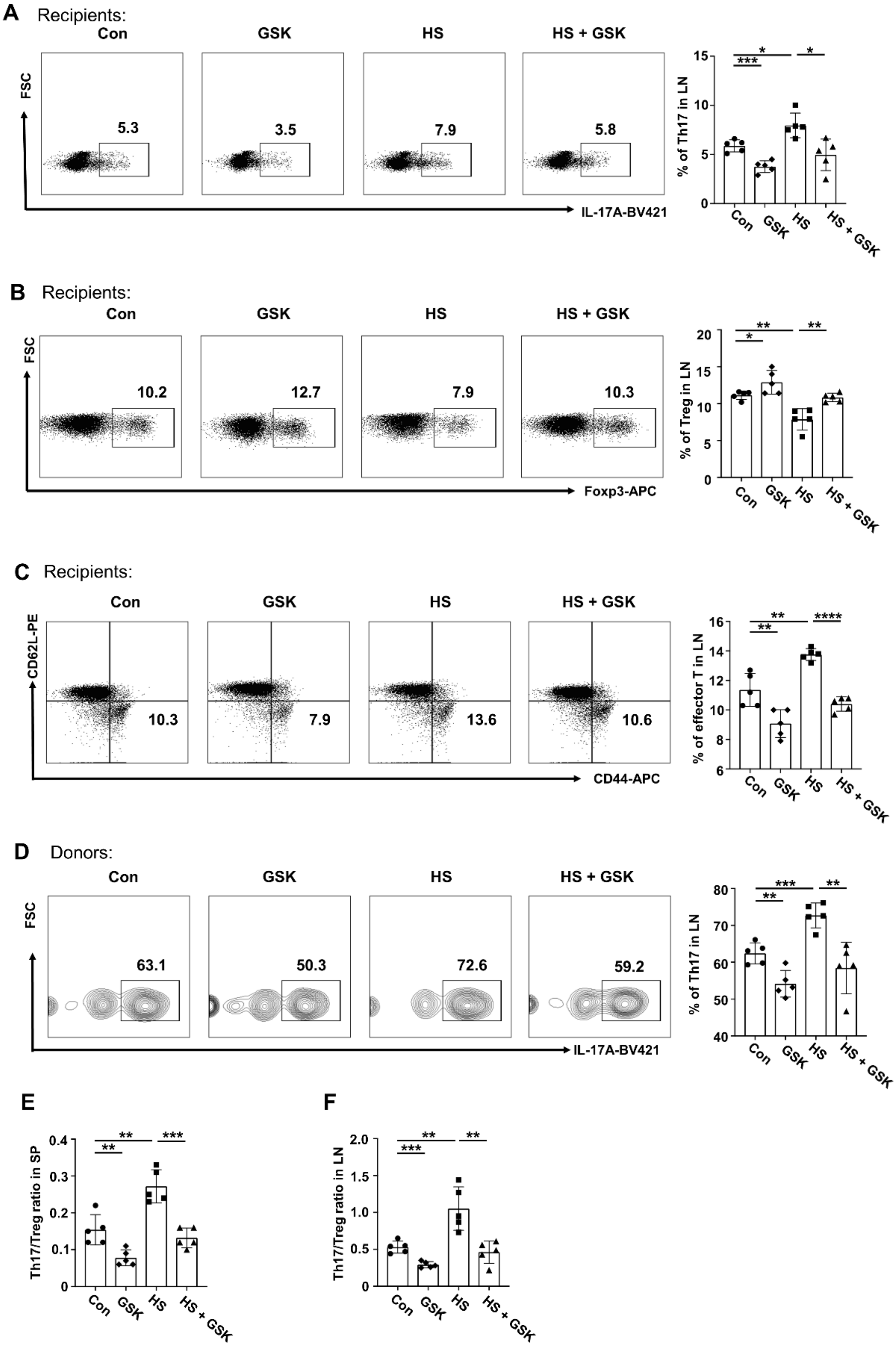

Figure S2. [Subpopulation analysis of CD4 T cells in lymph nodes of the adoptive transfer model], Related to Figure 3. (A) The proportion of Th17 cells within lymph node CD4 T cells of recipient mice (n = 5). **(B)** The proportion of Treg cells within lymph node CD4 T cells of recipient mice (n = 5). **(C)** The proportion of activated CD4 T cells within lymph node CD4 T cells of recipient mice (n = 5). **(D)** The proportion of IL-17A

producing donor cells in lymph nodes (n = 5). **(E)** The ratio of Th17/Treg cells within recipient-derived CD4 T cells in spleen. **(F)** The ratio of Th17/Treg cells within recipient-derived CD4 T cells in lymph nodes. Statistical significance was calculated by unpaired Student's *t*-test data are represented as mean ± SD. *p<0.05, **p<0.01, ***p<0.001, ****p<0.0001.

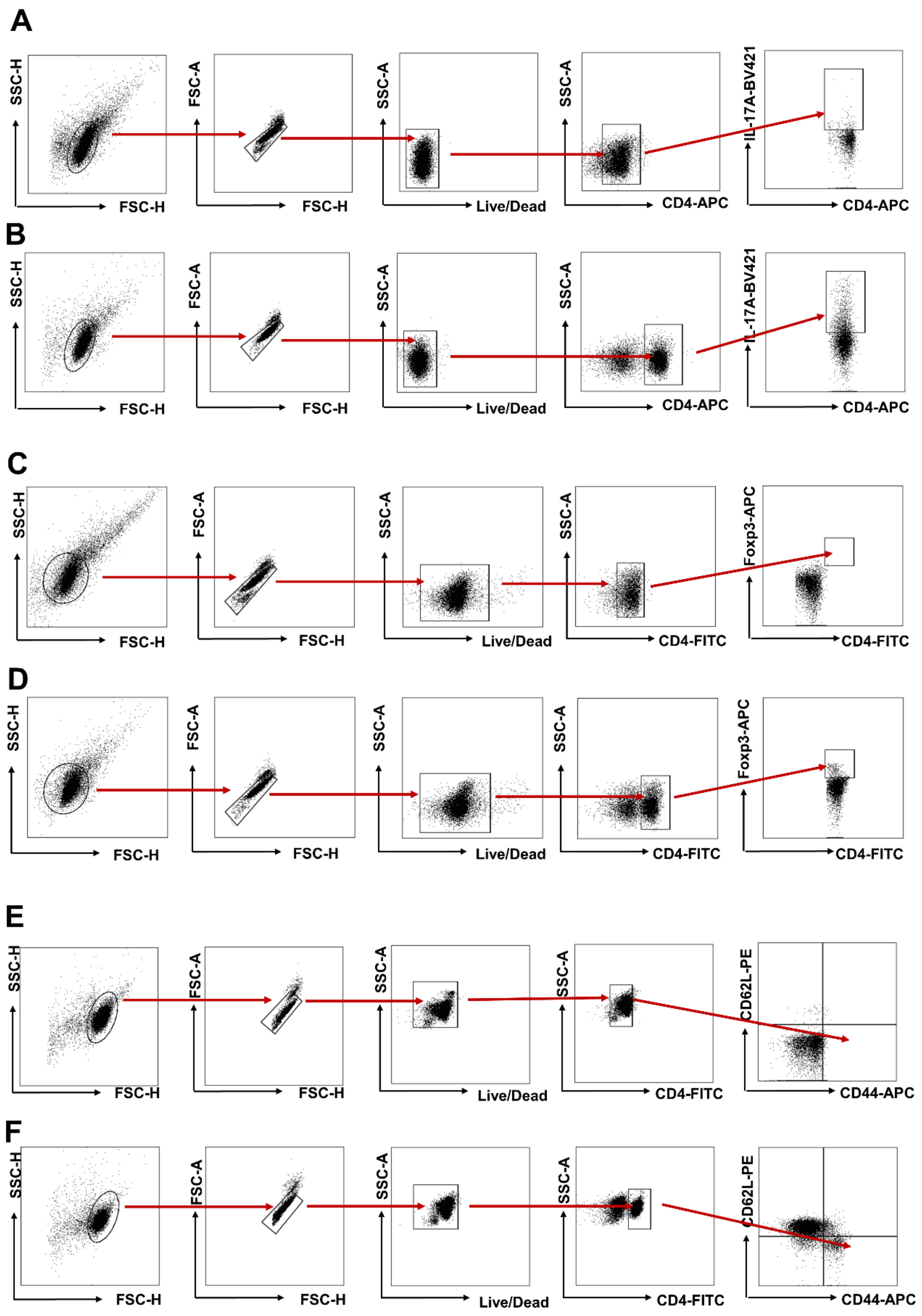

Figure S3. [Gating strategies for flow cytometry in CIA model], Related to Figure 5. (A). Gating strategy for Th17 cells. **(B)** Lymphocytes were initially gated, followed by the selection of live single cells and further gating on CD4 T cells. Within the CD4 T cell population, cells positive for IL-17A were gated to identify Th17 cells. **(C)** Gating

strategy for Treg cells. **(E)** Gating strategy for activated CD4 T cells. **(D, F).** Similarly, Foxp3$^+$ Treg cells and CD44$^+$ CD62L$^-$ activated CD4 T cells were gated.

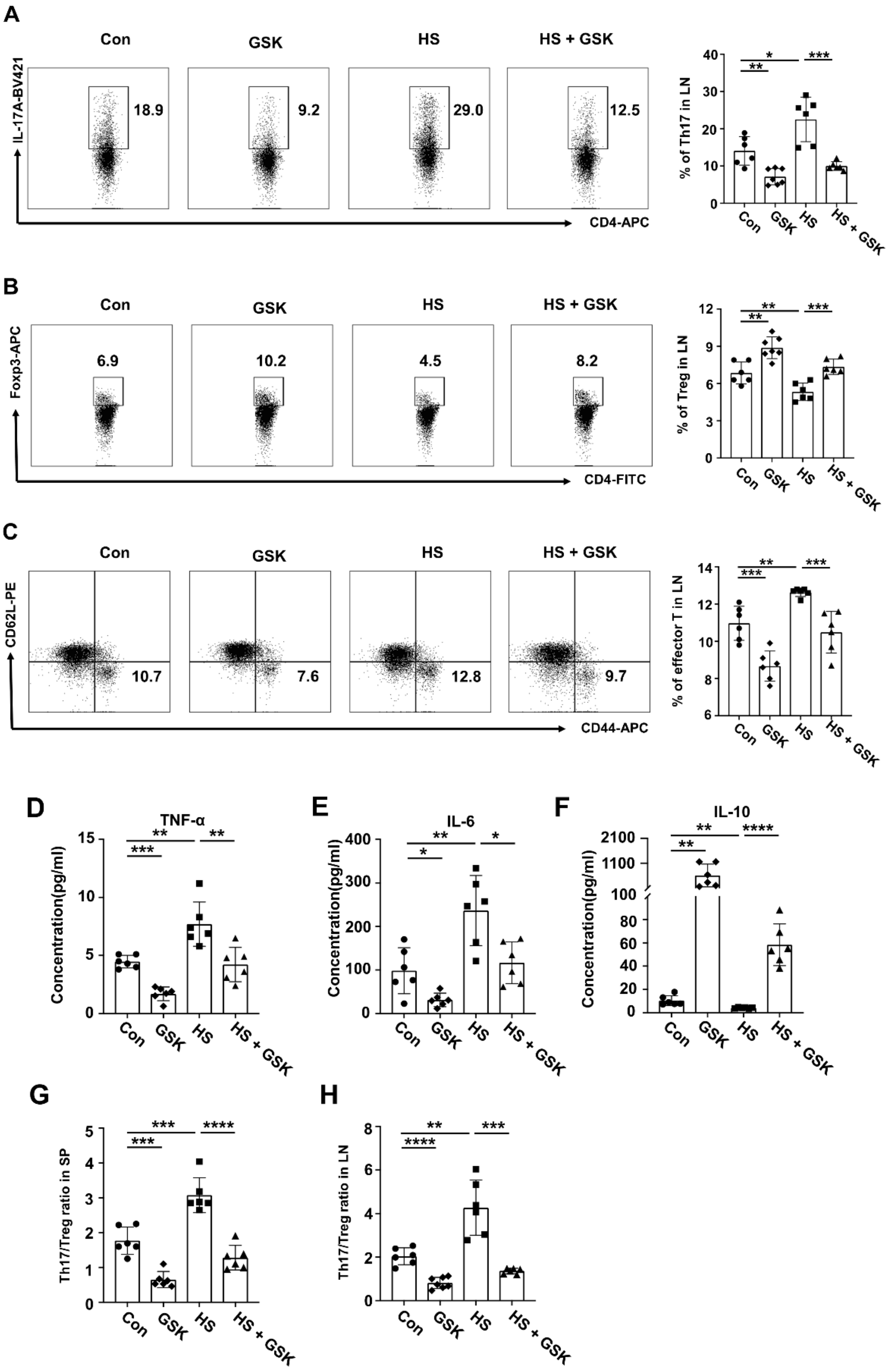

Figure S4. [Subpopulation analysis of CD4 T cells in lymph nodes and cytokine levels in the serum of CIA mice], Related to Figure 5. (A) The proportion of Th17 cells within lymph node CD4 T cells of CIA mice (n = 6). **(B)** The proportion of Treg cells within lymph node CD4 T cells of CIA mice (n = 6). (**C)** The proportion of activated CD4 T cells within lymph node CD4 T cells of CIA mice (n = 6). **(D)** The serum level of

TNF-α in CIA mice (n = 6). **(E)** The serum level of IL-6 in CIA mice (n = 6). **(F)** The serum level of IL-10 in CIA mice (n = 6). **(G, H)** The ratio of Th17/Treg cells in the spleen and lymph nodes of CIA mice. Statistical significance was calculated by unpaired Student's *t*-test data are represented as mean ± SD. *p<0.05, **p<0.01, ***p<0.001, ****p<0.0001.

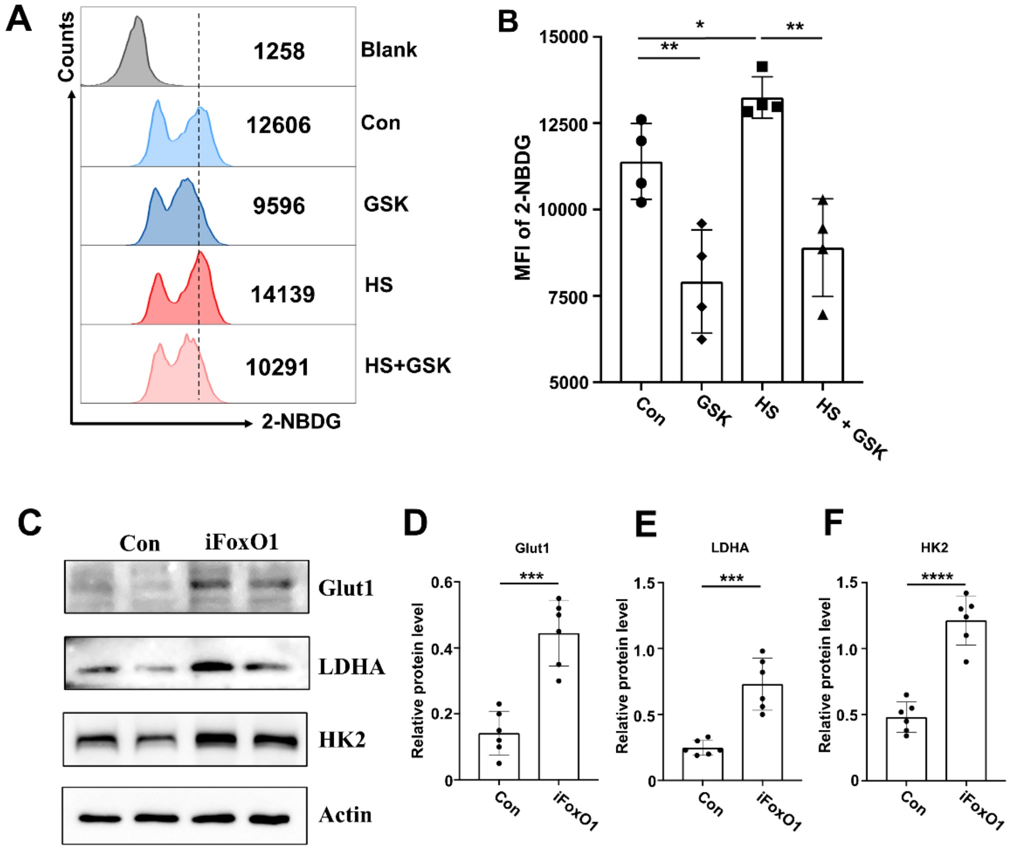

Figure S5. [Effects of GSK, 2-DG and iFoxO1 on glucose metabolism and Th17/Treg polarization], Related to Figure 6. (A, B) Glucose uptake indicated by the 2-NBDG signal intensity in CD4 T cells upon HS and/or GSK treatment (n = 3). **(C-F)** Western blot analysis of Glut1, LDHA, and HK2 in CD4 T cells upon iFoxO1 treatment. **(G, H)** Proportion of in vitro differentiated Th17 cells upon treatment with vehicle control (Con), 2-DG and iFoxO1 (n = 3). **(I, J)** Proportion of in vitro differentiated Treg cells upon treatment with vehicle control (Con), 2-DG and iFoxO1 (n = 3). Statistical significance was calculated by unpaired Student's *t*-test data are represented as mean ± SD. *p<0.05, **p<0.01, ***p<0.001, ****p<0.0001.
